# “*May we not orchestrate our own misfortune*”: a qualitative study on perception about causes and prevention of occupational injuries among bricklayers and carpenters in Osun State, Nigeria

**DOI:** 10.3389/fpubh.2025.1589458

**Published:** 2025-06-02

**Authors:** Temitope Olumuyiwa Ojo, Nisha Naicker, Funmilayo Juliana Afolabi, Adedeji Ayodeji Onayade

**Affiliations:** ^1^Department of Community Health, Obafemi Awolowo University, Ile-Ife, Nigeria; ^2^Division of Occupational Health, Faculty of Health Sciences, School of Public Health, University of the Witwatersrand, Johannesburg, South Africa; ^3^Department of Epidemiology and Surveillance, National Institute for Occupational Health, National Health Laboratory Services, Johannesburg, South Africa; ^4^Institute for Entrepreneurship and Development Studies, Obafemi Awolowo University, Ile-Ife, Nigeria

**Keywords:** occupational injuries, building construction, artisans, beliefs, perceptions, injury prevention

## Abstract

**Background:**

Occupational injuries (OIs) remain a major public health concern in the construction industry, especially in developing countries where underreporting poses significant challenges. The belief systems of construction artisans may shape their perceptions of workplace hazards and safety practices, yet these dynamics remain underexplored in Nigeria. Hence, this study explored the beliefs about causes and prevention of OIs among artisans in the informal sector of the construction industry in Osun State, Nigeria.

**Methods:**

Twelve focus group discussions (FGDs) were conducted in Osun State, Nigeria, with six FGDs each held with bricklayers and carpenters. Each FGD comprised five to seven participants. A semi-structured FGD guide was employed to moderate the discussions. Each session was facilitated by a moderator and a note-taker. Verbatim transcription was done for FGD audios, the transcripts were coded and thematically analysed using the ATLAS.ti software. Relevant direct quotations illustrating the themes and subthemes were cited accordingly.

**Results:**

Seventy artisans participated in the FGDs and all participants were male. The findings identified a range of beliefs about injury causation, with a strong emphasis on spiritual beliefs. These included notions of predestination by God and enchantment. However, a less common belief suggested that adherence to safety precautions could prevent injuries. Also, some participants expressed a nuanced combination of these perspectives. Perceptions of injury prevention were reflected in various subthemes, including the importance of prayers, avoiding conflicts, or taking jobs from others, maintaining safe housekeeping practices, government provision or subsidization of personal protective equipment (PPE) and its correct usage, provision of safety training and effective supervision.

**Conclusion:**

Workplace injuries among artisans were attributed to spiritual factors, limited access to safety equipment, and inadequate compliance with safety measures. Addressing these challenges requires culturally sensitive interventions, considering the perceptions of the artisans, alongside providing subsidized PPE, durable tools, targeted training, and effective supervision to foster a safer working environment and minimize injury risks.

## Background

The construction industry has consistently high rates of occupational injuries, posing a significant public health challenge and a pressing occupational safety concern (1, 2). This exceptionally high burden highlights the inherently hazardous nature of construction tasks and working conditions, making the construction industry one of the most dangerous sectors worldwide (3). In low-income countries the high injury burden in the construction industry is also related to the high rates of non-compliance with basic workplace safety measures. This challenge is further compounded by the chronic underreporting of incidents, especially in developing countries (4). Most injuries are never reported due to weak reporting systems, ignorance of the procedure, fear of job loss, or simply because of the informality of the employment arrangements for artisans in the industry with poor regulatory oversight (5, 6).

Bricklayers and carpenters are two primary artisan groups in informal sector of the construction industry that bear one of the highest burdens of occupational injuries (7). In sub-Saharan Africa, one-year occupational injury prevalence among construction workers range from 32.4 to 84.7% (8–10). Common injury types include lacerations, abrasions and puncture wounds while trips and falls—particularly from improvised scaffolds or ladders—constitute the leading mechanism of injury followed by crush injuries between materials or equipment (11, 12). In the absence of formal occupational health services or compensation schemes for informal workers, injured artisans bear substantial out-of-pocket medical expenses and income losses (13). The broader social and economic consequences; including chronic disability, reduced household income, and heightened poverty risk emphasize the urgent need for safety interventions to prevent occupational injuries in the informal sector of the construction industry (4, 13).

Establishing workplace safety controls are important in the construction industry, where injury burdens disproportionately affect artisans in the informal sector. These artisans, who are the main workers in most building construction projects in developing countries, often lack the specialized skills, training, and resources needed to protect themselves from the numerous hazards at work (14). Whereas their counterparts in the formal sector usually have structured safety programs, access to PPE, regular training, and effective supervision. Informal sector artisans often operate under precarious conditions and this makes them especially vulnerable to workplace injuries (2, 14).

Personal belief systems may shape workplace hazard perceptions and safety behavior among artisans in the informal sector of developing countries’ construction industry (15). The high rates of injuries, despite available interventions such as safety training and PPE, suggests that deeper cultural and attitudinal factors may undermine their effectiveness. Many artisans view workplace injuries as unavoidable, usually because of the level of insight and awareness regarding injury prevention (16). This view may be reinforced through poor supervision and the normalization of unsafe practices that are common in informal construction sites. This belief system and attitude barriers often mask the possible benefits of available safety measures, making meaningful change more difficult to achieve (15, 17).

Theoretical frameworks may help in understanding the nuances around safety behaviors and injury occurrence at workplaces. One theoretical framework that conceptualizes behavior as a product of interacting components is the beliefs, attitudes, subjective norms, and enabling factors (BASNEF) model. This model suggests that behavior is influenced by the interplay of individual beliefs, personal attitudes, social norms, and external enabling factors (18, 19). The BASNEF model has been shown to work in diverse settings, including a study among workers in an automobile plant where safety compliance was linked to beliefs, attitudes, norms, and knowledge (20) and among sawmill workers where beliefs about workplace hazards affected safety behavior (19). The model has also been applied in various other work settings (18, 21), the general populace (22, 23) and also used to explain patient adherence to medical treatment (24). The model suggests that the belief and attitude of an individual concerning a behavior, for instance, the use of PPE, are influenced by what he or she knows about it, previous experiences regarding the same, and the perceived outcome of the behavior.

Moreover, the model focuses on subjective norms, defined as the social pressures or the expectations perceived by individuals concerning certain behaviors (25). In many informal construction sites, unsafe practices are widespread, and safety standards are rarely enforced (15). This lack of oversight fosters an environment where compliance with safety protocols is often ignored and given less priority. Enabling factors, such as the availability of resources, training, and supportive policies, also play a very important role. Even when workers hold positive attitudes toward safety, their inability to act on these attitudes due to a lack of enabling factors such as affordable PPE or regular safety training and supervision further limits adherence to safety measures (26, 27).

Although, a few studies have been carried out on occupational injury prevalence and patterns among construction workers in Nigeria (7, 28–30), a gap exists regarding perceptions and beliefs among informal sector artisan construction workers (15). Due to the nature of work in the informal sector, these artisans often operate outside of safety regulations and frameworks (14). They could therefore perceive safety risks differently and this influences their safety behavior at work. Therefore, it is essential to explore the beliefs of artisans in the informal construction sector about the causes of workplace injuries and their views on effective prevention strategies.

Understanding the beliefs of artisans about the causes and prevention of occupational injuries is important for effective control. The present study has focused on these perspectives among artisans (bricklayers and carpenters) in Osun State, Nigeria. The study focused on bricklayers and carpenters because these two vocations bear the highest injury burdens in informal construction in Nigeria (7, 28). It is envisaged that study findings will provide useful insights that may inform targeted interventions which could reduce injury burden. This is important in fostering a culture of safety among this vulnerable workforce. It will not only improve the well-being of individual artisans but ensure that safe and decent work is promoted.

## Materials and methods

This qualitative study explores the experiences of bricklayers and carpenters in Osun State, Nigeria, as part of a broader research project on occupational injuries among building construction artisans (31). Osun State is one of the 36 sub-national territories, located in southwest Nigeria. The state has 30 local government areas (LGA) which are grouped into three senatorial districts (Ten LGA per senatorial district). The population of the state as projected from the last national census in 2006 is 5.6 million (32). The population comprises traders, artisans, farmers, and civil servants. Although the state has only a few large-scale industries, small-scale industries are more predominant and they include bakeries, bottle and sachet water production, and sawmills. Most informal artisans are in various trades across the state; they mostly operate singly while some work in small groups from shops or other work sites. These artisans include but are not limited to, plumbers, bricklayers, carpenters, auto technicians, spray painters, generator mechanics and tailors. They are usually organized in a self-managed association at the levels of zones up to LGAs, senatorial districts, and up to the state level (31).

### Participants and recruitment procedures

The study population comprised bricklayers and carpenters who work in Osun state and are registered with an association in the state. Bricklayers are craftsmen who use bricks and other masonry materials to construct walls and structures, aligning properly and bonding with mortar. Whereas carpenters typically work with wood to construct, install, and repair various fixtures and building structures. A total of 12 focus group discussions (FGDs) were held in different zones in Osun state. The selection was done as follows; Osun east senatorial district was selected out of the three in Osun state using simple random sampling (balloting). Among the 10 LGAs in the senatorial district, two LGAs (Ife Central and Ife East were selected). In each selected LGA, three artisans’ zones were selected out of 10 using simple random sampling (balloting). In each zone, two FGD sessions (one each) were held for carpenters and bricklayers. In each of the selected Local Government Areas (LGAs), all individuals engaged in bricklaying and carpentry were male, indicating a complete gender homogeneity within these occupational groups.

Each FGD session consisted of five to seven participants who met the inclusion criteria, which required them to be artisans aged 18 years and above with at least 1 year of experience in their vocation. However, artisans who were acutely ill and unable to respond to questions were excluded from the study. In all, a total of 70 artisans who met the study criteria were selected purposively and recruited into the study. Informed consent was obtained from all participants after explaining all study objectives and addressing any concerns or questions they had about the study.

### Data collection

A semi-structured FGD guide was designed and used to facilitate and moderate the discussion sessions. The focus group discussion guide included questions that explored artisans’ beliefs about the causes of workplace injuries and their perceptions of effective ways for prevention and control of injuries at work. The FGD guide was initially developed in English and subsequently translated into Yoruba, a local language most spoken in Nigeria. To ensure the accuracy and preservation of the original meaning of the questions, it was then back-translated into English. The translations were conducted by a linguistics expert. Each FGD session lasted between 40 and 65 min and was recorded using a tape recorder after obtaining consent to record. Before each session, the demographic characteristics of the participants were obtained and this included information on age, years of experience and level of education. The sessions were moderated by the first author and a trained note-taker. All FGD sessions were conducted in the Yoruba language and held in venues that ensured both visual and auditory privacy for participants.

### Data analysis

All audio recordings were transcribed verbatim in Yoruba and then translated into English language. The transcripts were checked and proofread by the translator, moderator and notetaker to ensure that they matched the recordings. The transcripts were then reviewed and coded in English language, using both deductive and inductive approaches. A codebook was populated using the set of codes derived from the transcripts. The Atlas ti software version 8 was used for coding and qualitative data analysis. Themes and subthemes were identified as they emerged from the data analysis. Throughout the manuscript, direct quotes from FGD participants were included in italics to illustrate themes and subthemes.

#### Credibility

The FGDs were conducted across six different sites with a fair representation of eligible artisans across the selected Local government areas. The first author moderated all discussions and conducted the analysis and therefore ensured that nuances were well captured through the entire study process. Digital tape recorders were used to record all discussions to ensure they were accurately captured and this was used for coding, rechecks and audits.

#### Transferability

To enhance transferability, the research process was clearly documented. Detailed information regarding the number of participants, the recruitment procedures, the duration of the sessions, and other relevant aspects of the FGDs was systematically recorded.

#### Dependability

The research methods were systematically documented, and an audit trail was kept for the data collection and analysis processes. This approach ensures that other researchers can replicate the study and therefore have the potential for comparable results.

#### Confirmability

To enhance confirmability, data analysis was done as a team by the researchers. This approach allowed multiple perspectives to be integrated into the analysis, enhancing the depth of interpreting findings. Anchoring the analysis in the experiences of participants reduces the threat of subjective biases affecting the data interpretation.

### Ethics and consent to participate

Informed consent was obtained from all participants. The study received ethical approval from the Human Research Ethics Committee (Medical) of the University of Witwatersrand, South Africa (M221066), and the Health Research and Ethics Committee of the Institute of Public Health, Obafemi Awolowo University, Nigeria (IPHOAU/12/1766). In addition, the study was conducted in line with the ethical standards as laid down in the Declaration of Helsinki and its later amendments or comparable ethical standards. Permission to carry out the survey was obtained from the head of the artisans’ association in the selected LGAs. Before participation, all respondents provided written informed consent after receiving an explanation of the study’s purpose, potential risks, and benefits. Participation was voluntary and confidentiality as well as data security was ensured. Respondents were informed of their rights decline or withdraw at any time without penalties.

## Results

Seventy artisans participated in the FGDs and all participants were male. The age of participants ranged between 24 and 70 years with 28 (40.0%) between 40 and 59 years. Their work experience varied from 4 to 49 years, with 27 (38.6%) having 11 to 20 years of experience. Slightly over half (39, 55.7%) of all participants had a secondary-level education (Table 1).

**Table 1:** Background characteristics of study participants.

Characteristic	Frequency (*n*)	Percent (%)
Age in years		
20–39 yrs	27	38.6
40–59 yrs	28	40.0
≥60	15	21.4
Highest educational attainment		
None	3	4.3
Primary	27	38.6
Secondary	39	55.7
Post-secondary	1	1.4
Years of experience as a construction artisan		
1–10 yrs	12	17.1
11–20 yrs	27	38.6
21–30 yrs	13	18.6
31–40 yrs	14	20.0
>40 yrs	4	5.7
Total	70	100.0

## Themes

### Perceptions of artisans about causes of workplace injuries

The belief of participants about injuries was explored regarding why injuries occur. A summary of the themes and subthemes are presented in Table 2. The subthemes that emerged from the discussion indicated that underlying the various beliefs was a strong perspective about spirituality, which included predestination by God, enchantment, the belief that injury or accident may be prevented if safety precautions are observed and a nuanced combination of these perspectives. This theme illustrates the interplay of cultural, spiritual, and practical influences driving the participants’ conceptualizations of injury causation and prevention.

**Table 2:** Themes, subthemes meanings from the focus group discussions on beliefs about causes and prevention of occupational injuries among construction artisans.

Theme	Subtheme	Subtheme meanings
Perceived causes of Occupational Injuries	Predestination by God	Injuries are often viewed as inevitable, predetermined by fate or supernatural forces.
	Enchantment	Injuries may be due to enchantments cast by perceived adversaries or vengeful clients.
	Por adherence to safety precautions	Injuries result from non-compliance with safety measures, such as the failure to use personal protective equipment (PPE).
	A combination of non-compliance to safety measures and spiritual factors	Injuries may result from an interplay of spiritual beliefs, workplace conditions, and personal factors.
Perceived ways of preventing occupational injuries	Prayers	Relying solely on prayers to God for protection was regarded as a potent method for preventing workplace injuries.
	Avoiding conflicts or taking up other artisan’s jobs	Refraining from taking others’ jobs to avoid potential enchantments that could lead to injuries.
	Practicing safe housekeeping and PPE use	The proper use of personal protective equipment (PPE) and adherence to safe housekeeping practices will reduce the risk of workplace injuries.
	Government has a role in safety training and supervision	The government could play a key role in injury prevention by providing subsidized PPE, standard equipment, and ensuring supervisory visits to enforce safety compliance.

#### Subtheme 1: occupational injuries are predestined and may be unavoidable

A subtheme which appeared prominent was the belief among many artisans that workplace injuries are not entirely avoidable or preventable and that injuries are expected to be part of everyday work routine. Furthermore, the notion that injuries are predestined—determined by fate or spiritual forces—was a recurring subtheme in the conversations.

*… all days are evil, there is no way to avoid getting injured whenever you go to work, but may we not encounter the injury that its impact will overwhelm us. There is nothing you can do about avoiding injury at work*. (P3, Carpenter, FGD 5)

*Workplace injury is not caused by (a) spiritual attack, it is God-ordained because (for instance) after you have tightened the wall bracket and you put a load on it and it suddenly collapses, this is not a spiritual attack but ordained by God.* (P2, Bricklayer, FGD 10)

#### Subtheme 2: occupational injuries may be due to enchantment

Another recurring subtheme was the belief that enchantments may underlie workplace injuries, particularly those that are severe or fatal. This subtheme was inductively categorized into two categories; the enchantments cast by fellow artisans whose jobs were taken, and those from disgruntled clients seeking revenge.

*Something happened not quite long after I got a job and another bricklayer went behind me to complete the job, if am not happy with the person I might attack the person spiritually such that it is either the person who went to hijack my job would either not feel well or get injured while the client will continue to spend money on the building every year, …” it is better for someone to snatch one’s wife than to for the person to hijack one’s job” because that is where one gets food to eat and another person went to hijack it, so if he (the aggrieved person) has the means, he might spiritually attack his colleague.* (P4, Bricklayer, FGD 8)

*…some people might take a job after being paid and abscond without doing the job, the client might place a curse on the artisan and the resultant effect might be that such an artisan might fall from a building*. (P1, Carpenter, FGD 9)

#### Subtheme 3: injuries at work are due to non-adherence to safety measures

Another subtheme, although less prominent was the notion that workplace injury is a consequence of poor adherence to safety precautions rather than spiritual causes or enchantments. This subtheme illustrates individual responsibility for control of workplace safety as an important issue to consider in preventing workplace injuries.

… *Workplace injury is not God-ordained; it is self-inflicted (preventable) as a result of carelessness. It is neither caused by witches nor wizards but it is self-induced. For instance, while working on a height and you discover that the wood you are standing on is not properly placed, instead of you to come down and adjust the wood, you will just say that you only have about 5 blocks to lay and you might get carried away that you will lay all your weight on the wood you are standing on, this might cause a fall.* (P3, Bricklayer, FGD 4)

*It is very important to protect yourself when you are at work. Often when you are laying a foundation with stones, the more you hit the stones with a hammer, the more the stones will splinter into fragments, so if you are wearing a short sleeve clothes and you are not wearing gloves and other protective wear, the pieces of stones will tear the skin and blood will start gushing out*. (P5, Bricklayer, FGD 10)

*Injury at (construction) site is not a curse, it is not spiritual but it is self-induced, May we not orchestrate our own misfortune. It is not caused by any curse laid by anybody*. (P5, Carpenter, FGD 6)

#### Subtheme 4: the combination of both spiritual factors and poor adherence to safety measures may contribute to injury causation

Some of the participants believed that spiritual factors, as well as non-compliance with established safety protocols, may have a synergistic effect that could contribute to workplace injuries. This perspective underlines the possibility of an interplay between spiritual factors, enchantments and work-related behaviors. This subtheme also demonstrates that artisans could look at the elements as not being mutually exclusive but can be possibly combined to cause injuries.

…*We have earlier explained the self-induced aspect. For instance, a person who left home and went to take alcohol before going to work, there is no way he can be engaged in any work that he won’t get injured, although in our profession some injuries are caused by spiritual, but most of the injuries are self-induced because when you drink alcohol before coming to work, you won’t be able to see clearly when working and you might get injured at any time while working.* (P4, Bricklayer, FGD 2)

### Perceptions about injury prevention

The main theme on perceptions on injury prevention was supported by various subthemes which include the importance of prayers, proper housekeeping practices, avoid taking away jobs from others or creating conflicts, Government provision/subsidized PPE and its proper use as well as continuous safety training, and proper supervision.

#### Subtheme 1: the perceived role of prayers in preventing occupational injuries

Many participants in the study expressed the belief that praying to God for protection was an effective and essential method of preventing workplace injuries. Prayer was perceived as the only reliable option for ensuring personal safety. The belief emphasizes acceptance of hazards as part of daily work routine, with the hope that divine intervention will prevent life-threatening injuries.

*There is nothing we can do to prevent it, we all know that all days are evil and workplace injury is unavoidable, may we not experience the injury that will claim our lives and may God continue to protect us*. (P6, Bricklayer, FGD 5)

*We should still continue to pray for God’s protection which is the ultimate*. (P5, Bricklayer, FGD 1)

*What I will say is that we should all be prayerful … that God should protect us in our place of work.* (P1, Carpenter, FGD 7)

#### Subtheme 2: avoiding job conflicts so as to avert injuries attributed to enchantments

Given the widespread belief in spiritual influences on occupational injuries, many artisans consider it important to avoid taking other people’s jobs to avoid enchantments that may evoke injuries at work. Seeing that these beliefs can create the potential for conflict, the artisans’ association has put in place a mediation process. Where there are controversies over job assignments or encroachment, for example, the association intervenes to mediate, aiming to settle the matter amicably and prevent members from resorting to enchantment or laying a curse on one another.

*I think the most effective preventive measure (in this area) is to avoid hijacking another person’s job. We always sound this note of warning to the apprentice during their freedom ceremony. We are all different from each other, if you hijack a person’s job, he may not be bothered and he will surrender everything to God, but if you do that to another person he may not take it lightly*. (P6, Carpenter, FGD 12)

*If I take up a job … and another person now takes over the job, this may claim the life of the person that takes over the job. I have seen it before but nowadays the association has started regulating all the lapses which is the essence of the association. People now lodge complaints to the association regarding this to avoid inflicting injuring on others and the (artisans’) association addresses the situation and justice will be served accordingly*. (P3, Carpenter, FGD 5)

#### Subtheme 3: the perceived role of government in supervision, training and providing some PPE as well as standard equipment

Many participants stressed that the government has a vital role in reducing workplace injury. They highlighted the need for subsidies to ensure access to personal protective equipment (PPE) like helmets and gloves, as well as high-quality tools and machinery, e.g., metal scaffolds, particularly for individual workers. Participants also stressed the necessity of regular supervisory visits to worksites for both enforcement of safety regulations and to educate workers on the best practices of preventing injuries.

*Just like my colleague mentioned about the use of personal protective equipment, we can’t afford to buy them because we are not making much money, and we use the little money we are making to feed. So, if the government can provide us with personal protective equipment occurrence of occupational injury will be reduced*. (P4, Carpenter, FGD 7)

*I think that the government can appoint a task force that will supervise and caution people who are treading a dangerous path by not using PPEs. I have seen that while working in the company, the task force will supervise the workers*. (P2, Bricklayer, FGD 2)

*… the government can intervene in the issue of wall bracket that we use for our work. Those of us who are not financially okay normally use bamboo for scaffolding when working on a 5 to 6-storey building, but those people who are ‘financially okay´ use metal ladders to climb heights. If the government can provide the metal ladder for us or even rent it out to those of us who would not be able to afford to buy it. So we need the government to assist in providing the metal scaffolding for us*. (P2, Bricklayer, FGD 10)

*I already mentioned that even if they (the government) give us these PPEs for free, not everyone can use them because we are not used to them, we need to be trained*. (P6, Carpenter, FGD 5)

#### Subtheme 4: PPE use and safe housekeeping

Participants emphasized that the proper use of personal protective equipment (PPE) and adherence to safe housekeeping practices are important for preventing workplace injuries in construction sites. They also expressed that it is important to maintain an organized, clean work environment as this may reduce such risks such as slips, trips, and falls, which are common causes of injury.

*Some people will be fully kitted with safety items such as boots when going to work that you will think that they are soldiers, when such people fall, they won’t get injured*. (P2, Bricklayer, FGD 1)

*There is no way you can prevent injury other than being mindful of your environment, being careful is the most important measure to prevent injury at work. Whether you are working in the workshop or outside the workshop, you must be careful to prevent injury*. (P6, Carpenter, FGD 7)

## Discussion

Every worker should enjoy the best level of health and a decent, safe work environment, which will translate to maximum productivity and economic development (3). These ideals closely align with the core objectives of the eighth SDG (33), emphasizing the need to focus on informal work settings in developing countries like Nigeria, where such contexts are often neglected and deserve greater attention to improve safety and well-being (34). The present study has investigated the perceptions of informal construction artisans concerning the causes and prevention of workplace injury. It is perhaps one of the few studies on this prominent issue in the Nigerian context. Understanding such perceptions is necessary for developing focused interventions that would reduce the prevalence of occupational injury, improve workplace safety and, thereby, enhance productivity and well-being in the informal sector.

### Artisans’ beliefs about causes of workplace injuries

From this study, it was established that the belief in spiritual dimensions underlying injury causation was widespread among artisans. Many respondents believed that injuries were pre-ordained or allowed to happen by supernatural forces and are therefore inevitable and may not be preventable by humans. These kinds of beliefs reflect a loss of personal agency or locus of control among artisans and may encourage passivity, dissuading active adoption of workplace safety precautions. This is not surprising, considering that the Nigerian society and indeed many African societies have deep roots in spiritual traditions and beliefs (16, 17, 35). A similar finding was reported among other groups of artisans in other African countries who believed that spiritualty and witchcraft practices may underpin injury causation (35). In such a cultural context, perception and behavior are moulded, and events are often given spiritual explanations (15, 35). It is essential to consider this perspective when designing and implementing safety interventions for artisans to ensure their relevance, acceptance, and maximum uptake.

Another important factor that may cause injury as reported by artisans in this study was related to poor adherence to workplace safety measures. However, other participants agreed that a combination of spiritual factors and disregard for safety precautions can result in workplace injury. Important issues identified under non-adherence to safety precautions included substance use at work, poor housekeeping practices, and failure to use PPE among others. These have been cited in previous studies with similar contexts (28, 30). This finding highlights the need for targeted interventions addressing substance use management, proper PPE use, and other personal and psychosocial factors that contribute to workplace injury risk. These components should be incorporated while designing interventions aimed at promoting a culture of safety among informal sector artisans in building construction.

### Artisans’ perceptions about injury prevention

From this study, many artisans held the belief that workplace injuries often had spiritual underpinnings, and they offered spiritual strategies for preventing injury. Therefore, recourse to prayer became one of the primary and often apparently trusted ways to guard against occupational injuries. For many artisans, prayer is not only a spiritual practice but also a very effective way of protection that goes beyond any physical safety measures (17). This finding aligns with reports of some past studies conducted in similar contexts where prayers were said to prevent injuries before working at construction sites (36, 37). Such a belief represents a deep spiritual connection in the community, thus meaning that these perspectives need to be considered and put in context when promoting workplace safety behavior among artisans.

In addition, conflict mediation among fellow artisans was recognized as a means of preventing enchantments that may potentiate injuries through supernatural means. This belief, while steeped in spirituality, may not align with logical or scientific explanations of workplace injury causation. It is, however, of key importance to the artisans and is inherently part of their belief system. This conviction is so pronounced that artisans’ associations have undertaken proactive measures to mediate conflicts, especially those about competition for construction jobs, such as usurping another person’s work. By doing this, they seek to prevent aggrieved members from turning to supernatural means to inflict workplace injury spells on their rivals. Acknowledging and respecting these beliefs is important while planning context-specific interventions to promote safe working environments and prevent injuries among these vulnerable artisans.

Another important aspect is the responsibility of the government, as well as other stakeholders, to provide personal protective equipment at subsidized costs, among other safety tools needed in the workplace. This is important because even when workers are exposed to hazards, the chances of injury are minimized once proper equipment is available and applied. For instance, the availability of durable metal ladders and scaffolding can help avert falls, one of the most common types of injuries among bricklayers and carpenters. This is quite relevant in Nigeria and other developing countries where artisans depend on wooden ladders and rickety bamboo scaffolds during the construction of buildings, thereby increasing their vulnerability to accidents (38, 39).

The BASNEF model assumes that health-related behaviors arise from the interactive influences of Beliefs, Attitudes, Subjective Norms, and Enabling Factors. In our focus groups, most artisans attributed work-related injuries to spiritual factors—predestination or enchantment—thus illustrating the beliefs component by viewing injuries as an unavoidable fate and thus reducing their perceived control over safety. In contrast, a smaller number of participants recognized that observing safety precautions could avert harm, reflecting the attitudes domain and shows a willingness to adopt behavior change when the benefits of safety measures are clear. Narratives that highlight the influence of respected peers and artisans’ leadership illustrate subjective norms, emphasizing that shared expectations and peer leadership can either encourage risk-taking or support safety habits.

Key enablers identified by artisans were state of availability of personal protective equipment and a need for hands-on, experiential training. These findings highlight that, even in situations where attitudes and beliefs align with safety behaviors, the absence of tangible resources and institutional support can limit the adoption of protective behaviors (40). Addressing these challenges using frameworks-grounded solutions is essential to transform behavioral intentions into sustained actions for safety improvements among artisans.

To enhance the feasibility and sustainability of the interventions proposed for safety, a multi-stakeholder approach is recommended. Specifically, a public–private partnership model is suggested, where government agencies collaborate with artisan associations and civil society organizations to enable local production and equitable distribution of personal protective equipment (PPE) and other essential work tools at subsidized rates. This strategy will address cost constraints to PPE adoption and promote local economic development through community-level production programs. Existing institutional structures, e.g., local council government town planning inspectorate units can take up the role of coordinating regular on-site inspections and monitoring PPE use among construction artisans.

Furthermore, much emphasis was placed on the importance of training and supervision. Workers must be specifically trained on how to effectively use personal protective equipment while at work on construction sites. Similarly, the role of a supervisor should include confirming that artisans adhere to safety measures at work. This agrees with other works that identified training and constant supervision as ways to foster an appropriate safety culture that could prevent workplace injuries (34, 39).

As regards the study limitations, a qualitative design was employed and this explored perceptions and experiences in-depth but did not produce findings that are statistically generalizable. Similarly, this study was conducted in only one state: Osun State, Nigeria, thereby limiting the applicability of the study findings to other regions with different contexts. In addition, the study was conducted among bricklayers and carpenters, therefore the results may not be generalizable to all artisan groups in the informal sector of the construction industry. There may be differences in attitudes among registered construction artisans and those who are not members of registered associations. However, it is expected that there will be few unregistered individuals in this instance, as most artisans tend to join associations to access the benefits they provide. These limitations should be noted when interpreting the findings or attempting to generalize these in broader settings or populations.

Future studies may explore how cultural beliefs, such as predestination, shape the adoption of safety practices among construction artisans. Also, participation from a greater representation of diverse subjects, including independent and unregistered artisans, would enhance generalizability and provide broader insights on safety behavior across the construction industry.

## Conclusion

Workplace injuries among artisans in this study were perceived to be influenced by the combination of poor safety measures, inaccessibility to appropriate safety equipment, and deeply rooted cultural and spiritual beliefs. Addressing these challenges requires a multifaceted approach that considers both practical and contextual factors. The provision of subsidized personal protective equipment and durable construction tools, such as metal ladders and scaffolding, is relevant in the reduction of exposure to hazards and falls, among other common injuries.

Training and supervision are also important in promoting appropriate use and adherence to safety procedures. Workplace safety will be enhanced by artisan-focused, safety training that is regularly monitored. Moreover, recognizing the cultural and spiritual beliefs about injury causation is vital for designing interventions that resonate with artisans’ beliefs and encourage adoption of workplace safety measures. Together, these interventions can be used to create a safer and more productive work environment for informal sector artisans.

## Data Availability

The raw data supporting the conclusions of this article will be made available by the authors, without undue reservation.
