# Correction: “*May we not orchestrate our own misfortune*”: a qualitative study on perception about causes and prevention of occupational injuries among bricklayers and carpenters in Osun State, Nigeria

**DOI:** 10.3389/fpubh.2025.1665543

**Published:** 2025-08-05

**Authors:** Temitope Olumuyiwa Ojo, Nisha Naicker, Funmilayo Juliana Afolabi, Adedeji Ayodeji Onayade

**Affiliations:** ^1^Department of Community Health, Obafemi Awolowo University, Ile-Ife, Nigeria; ^2^Division of Occupational Health, Faculty of Health Sciences, School of Public Health, University of the Witwatersrand, Johannesburg, South Africa; ^3^Department of Epidemiology and Surveillance, National Institute for Occupational Health, National Health Laboratory Services, Johannesburg, South Africa; ^4^Institute for Entrepreneurship and Development Studies, Obafemi Awolowo University, Ile-Ife, Nigeria

**Keywords:** occupational injuries, building construction, artisans, beliefs, perceptions, injury prevention

An incorrect Funding statement was provided.

Old statement is: “The author(s) declare that financial support was received for the research and/or publication of this article. This study was supported a grant for PhD research provided by Consortium for Advanced Research Training in Africa (CARTA) which is funded by the Carnegie Corporation of New York (Grant No–B 8606.R02), SIDA (Grant No: 54100029) and the Developing Excellence in Leadership, Training and Science in Africa (DELTAS Africa) Initiative (Grant No: 107768/Z/15/Z). The views expressed in this publication are those of the authors and not necessarily those of the partners in the consortium”.

The correct funding statement reads:

“The author(s) declare that financial support was received for the research and/or publication of this article. This research was supported by the Consortium for Advanced Research Training in Africa (CARTA). CARTA is jointly led by the African Population and Health Research Center and the University of the Witwatersrand and funded by the Carnegie Corporation of New York (Grant No. G-21-58722 and G-PS-23-60922), SIDA (Grant No: 16604), Norwegian Agency for Development Cooperation (Norad) (Grant No: QZA-21/0162), Oak Foundation (Grant No. OFIL-24-091), and the Science for Africa Foundation to the Developing Excellence in Leadership, Training and Science in Africa (DELTAS Africa) programme (Del-22-006) with support from Wellcome and the UK Foreign, Commonwealth & Development Office and is part of the EDCPT2 programme supported by the European Union. The statements made and views expressed are solely the responsibility of the authors.”

The original version of this article has been updated.

